# Online or face-to-face instruction? A qualitative study on the electrocardiogram course at the University of Ulm to examine why students choose a particular format

**DOI:** 10.1186/s12909-017-1053-6

**Published:** 2017-11-09

**Authors:** Oliver Keis, Claudia Grab, Achim Schneider, Wolfgang Öchsner

**Affiliations:** 1 Medical Faculty, Office of the Dean of Studies, University of Ulm, Albert Einstein Allee 7, 89081 Ulm, Germany; 2 Center for Surgery / Department for Cardiac Anesthesiology, University Hospital Ulm, Albert-Einstein-Allee 23, 89081 Ulm, Germany

**Keywords:** ECG course, E-learning, Face-to-face teaching, Advantages and disadvantages, Flexible time management, Flexible location, Interaction possibilities, Commitment, Extrinsic and intrinsic motivation

## Abstract

**Background:**

Since the introduction of the e-learning electrocardiogram (ECG) course ‘ECG Online’ into the curriculum at the University of Ulm, a small but relatively constant number of students have decided not to participate in the online course but to attend the face-to-face course, although the content of both courses is identical. The present study examined why students prefer one format or the other.

**Methods:**

In a qualitative research approach, ten medical students were questioned in a guided interview. At the time of the survey the interviewees were enrolled in the 7th to 10th semesters. Among the respondents, 2 had participated only in the face-to-face ECG course, 4 only in the online version and 4 in both the face-to-face and the online course.

**Results:**

Interestingly, the very factors associated with e-learning – and always praised as advantages of it – are viewed critically by the students. Thus, although the 24-h access to learning content was consistently evaluated positively, the unlimited availability (lack of expiry date) was not seen as conducive to learning. The lack of fixed time constraints and the attendant lack of pressure were important reasons why some of the students had discontinued the online course prematurely. A similar distinction was seen in the flexibility of location for e-learning, because the very obligation to be physically present on a particular day at a fixed time led to a higher degree of commitment to courses and a willingness to actually attend the course until the end. In addition, if the content has a high degree of perceived professional relevance face-to-face courses are preferred because they offer the possibility of direct interaction.

**Conclusions:**

Even though the small sample size limits the generalisability of the results, our findings indicate that when developing online courses students’ needs could be better met if measures were included to strengthen extrinsic and intrinsic motivation and formats were favoured that enable students to have a minimum level of personal interaction with the lecturer.

## Background

The increasing introduction of e-learning formats into medical education has resulted in many studies; numerous authors have thereby established that in medical education the learning outcome through e-learning formats is at least equal to that of traditional methods [1, 2] or that e-learning contributes additional positive effects to traditional methods [3, 4]. In addition to the findings of these outcome-oriented studies, and of more relevance from the perspective of educational planners, the following advantages of e-learning formats have been cited in the literature: support of individual and community learning, access to a high number of students, greater flexibility in learning through personal choice of time and location and good availability and cost-effectiveness [5–8]. However, possible disadvantages of certain e-learning formats have also been shown recently. In her study on economics students, Wigger determined that e-learning students show poorer professional competence than conventionally taught students. In this study, students evaluated working independently, which they are unaccustomed to, and the time required as negative [9].

Because numerous studies have focussed on the outcome of e-learning courses and the perspective of educational planners, in the current study we decided to look at a different perspective, i.e. that of the students. Teaching courses are usually offered as either a face-to-face or an online course or in a blended learning format. However, our university offers a special configuration: one of the elective courses at the Faculty of Medicine at the University of Ulm, the electrocardiogram (ECG) course, is offered to all medical students in the 7th to 10th semesters as an e-learning and face-to-face course. The subject matter of both formats is identical. Students are free to choose either format or both, although not simultaneously; the choice of format, or the choice not to take this elective course, has no effect on the progress of students’ studies. The course communicates basic information about the resting ECG and the interpretation of ECG curves. Experience has shown that about half the students in semesters 7 to 10 attend one of the two formats of the ECG course during the course of their studies. On average, about 12 students enrol for the face-to-face course per semester, while approximately 160 students participate in the online course.

### Study aim

We used the fact that the ECG course is offered in two different formats to examine the following questions from the students’ perspective: (1) What are the reasons why students choose the face-to-face or online format of a course when the content of both formats is identical? (2) What conclusions are to be drawn from these preferences for the design of e-learning formats that better meet students’ needs? We hoped that the availability of one course in two different formats and the possibility to examine the students’ perspective would allow us to obtain first-hand insights to support the further development and design of our e-learning tools.

We decided to focus on the students rather than outcome-related criteria, such as examination results, to obtain information on students’ subjective perspectives and opinions on the two formats.

## Methods

### Participants

In January 2015 we e-mailed all medical students in the 7th to 10th semesters at the University of Ulm who took the online (*N* = 317) or face-to-face (*N* = 23) ECG elective course during the summer semester 2014 or the winter semester 2014/2015 (*N* total = 340). In the corresponding periods, a total of 674 students were enrolled in semesters 7 to 10.

We were able to recruit 10 students into the study (3 males; 7 females). Students attended the 7th (*n* = 1), 8th (*n* = 2), 9th (*n* = 5) and 10th (*n* = 2) semester, respectively.

All participants had previously participated in at least one curricular computer-assisted learning programme as part of their course. Furthermore, all interviewees stated that in both their university and private environments they met the technical requirements necessary for participating in an online course.

Participants provided written informed consent after being assured that their data would remain anonymous for data analysis. They received € 30.00 for participating in the study.

The ethics committee of the University of Ulm confirmed that no approval was required for this study.

### Curriculum

The online ECG course consists of 12 audio visual lessons of about 1 h duration and 2 to 4 online exercises (usually ECG interpretations) that are provided for each lesson. The aim of the course is to learn about the basics of the resting ECG and its interpretation for clinical purposes. Once a learning unit has been completely worked through (including the exercises) the next lesson is released (including sample solutions for exercises). For questions, students may contact the course instructor per e-mail. Completed e-learning lessons are available at any time to all students participating in the online course to allow for practise and repetition.

The content of the face-to-face course is identical to that of the online course. It consists of 12 lectures (90 min each) dealing with the basics and interpretation of the resting ECG and ECG curves. Students participating in the face-to-face course receive detailed notes on each lecture and have access to the lecture slides via the university learning platform.

After completing the full course, students can take a voluntary written final examination. If they pass the examination, they receive a certificate of participation (optional ECG certificate). This examination is summative and held jointly for all students at the end of the course, independent of the chosen course format. The mark scheme is shared with students. Though dropouts and assessment performance are not routinely documented, on average, about 25% of the students who enrol in the online course take the examination.

### Data collection

We used a qualitative research approach to investigate the reasons why students had chosen the online or face-to-face format of the course. We chose a semi-structured interview as the evaluation procedure because, in addition to allowing open questions and answers, it also ensured comparability of the interviews [10].

The interview questions were compiled by means of the SPSS method of Helfferich [11] (SPSS stands for the German words ‘sammeln, prüfen, sortieren, subsumieren’, i.e. collect, check, sort, prioritise); the resulting interview guide consisted of 12 questions (see Table 1).

**Table 1:** Interview questions with category clusters and superordinate topic areas

Superordinate topics	Category clusters	Allocated questions
General strengths and weaknesses of the two formats	Reasons for and against the online and face-to-face formats	4. What do you see as the advantages or disadvantages of e-learning formats compared to a face-to-face format? 11. What general features do you think an ideal online course has to have? 12. What would you expect in general from an ideal face-to-face course?
	Choice of format from the perspective of education planning	6. If you were an education planner, which courses do you think should be taught online, which in a face-to-face format?
	Prerequisites for changing behaviour because of the format chosen	5. Which conditions would need to be created so that you would participate in the ECG online or face-to-face course?
Personal reasons for choosing a particular format	Content-related factors	1. What motivated you to learn about the topic ‘ECG’ during your studies? 2. Which factors make it easier or more difficult for you approach the topic ‘ECG’?
	Learning style factors	7. How would you describe your learning style? 8. Which teaching methods do you prefer during your study of medicine? 9. Can you think of situations in your studies so far in which the exchange with fellow students and lecturers was important for your learning success? 10. If you want to learn a particular subject matter, how do you ideally manage your time for learning?
	Personal reasons for making a particular decision	3. On what criteria did you base your decision to participate in the online or face-to-face course?

Two interviewers conducted the 20- to 30-min interviews according to recommendations in the literature [12]. The interviewers were not involved in teaching the ECG course, nor were there any dependencies between interviewers and interviewees. All interviews were recorded on tape and subsequently transcribed and anonymised. In accordance with Lamnek [13], transcriptions were conducted by two independent assistants who had not been involved in the interviews and who were trained beforehand.

In addition, study participants were sent a brief online questionnaire asking about the technical prerequisites for online courses (e.g. ‘Do you think the university’s technical equipment is adequate to allow you to participate in online courses?’) and the assessment of the students’ own e-learning competence (‘How do you rate your competence in dealing with e-learning formats?’). Participants were also asked to specify their sex and current semester and whether they had their own computer equipment.

### Data analysis

The interviews were evaluated following the qualitative content analysis according to Mayring [14].

Interview responses were clustered into 6 categories, which in turn were assigned to the two superordinate topic areas ‘General advantages and disadvantages of the two formats’ and ‘Personal reasons for choosing a particular format’ (see Table 1). The first topic area required the students to give their perspective on a higher level of abstraction, whereas the second reflected their individual perspective.

Although we collected both qualitative and quantitative data, the present paper focuses on the qualitative findings. Quantitative data are supplementary and will be mentioned as appropriate in the sections Discussion and Conclusion.

## Results

Among the interviewees, 6 had participated in the face-to-face course and 4 in the online version; 2 of those who participated in the face-to-face course had previously completed the online course and 2 had previously discontinued it. Thus, a total of 8 interviewees could provide information on the online course and 6 on the face-to-face course.

### General strengths and weaknesses of the two formats

#### Reasons named by students for and against online and face-to-face formats

Many of the advantages and disadvantages of the two formats listed in Table 2 have already been discussed elsewhere [15]. The fact that three respondents referred to the supportive character of the face-to-face courses is therefore all the more interesting. Thus, the fixed location and time of this format were experienced as positive because they made students commit to participating and made them study the topic.

**Table 2:** Mentions by students (*N* = 10) of reasons for and against online and face-to-face formats and of desired improvements in the two formats

	Pro (number of mentions)	Con (number of mentions)	Desired Improvements (number of mentions)
Online	Flexible time management (8) Individual learning speed (4) Repeatability of the content (4) Flexible location (2)	Lack of opportunities to interact (6) Requires more initiative/self-discipline (4) Takes up more time than the face-to-face course (1)	Integration of exercise or control tasks (7) Personal interaction with direct contact person (4) Integration into the compulsory curriculum (2) Integration of an expiry date to accessing the course (1) Integration of blended learning elements (1)
Face-to-face	Opportunity to have direct interaction with the lecturer (6) More attention in small group teaching (6) More commitment to participate and studying the topic more closely than in the online format (3)	Effort of participating (travel times, fixed location, overlapping with other courses, etc.) (1)	Integration of patients into the course (1) Optimizing timetables to avoid time gaps between courses (1) Reducing group sizes to promote active participation during the sessions (1)

In addition, presence of the lecturer was perceived as increasing attentiveness, especially when the subject matter was difficult. The prospect of being asked directly in the face-to-face course, but not being able to answer, motivated students to adequately study the course topics.

*‘[...] face-to-face with the lecturer, of course you don’t really want to lay yourself open (breathes in) [...] so then I always try [...] so always, always try to be up to date [...].’* (Interview 9, authors’ translation)

This effect could be strengthened by the attention in small group teaching, which is much more beneficial for attentiveness, not least because there are ‘*fewer possibilities to hide’* (Interview 7, authors’ translation) (6 mentions).

This finding also corresponded with several statements that the greater necessity of self-initiative or self-discipline in the online course was perceived as negative (4 mentions), as was the lack of interaction possibilities (6 mentions).

Therefore, when considering the question of the ideal characteristics of online courses, the integration of exercise or control tasks (with feedback) (7 mentions) and the opportunity for personal interaction with a direct contact person (4 mentions) play a decisive role. An assessment of course participation (e.g. in the form of a mark) would also add value and thus ultimately increase motivation on the part of the students.

With regard to time structure, one respondent’s comment is noteworthy: the student stated that in an ideal online course the actual central element of online courses, i.e. the ability to access the learning content as often and for as long as desired, would have to give way to a limit on the time that the material can be accessed. The ‘*time constraint*’ (Interview 2, authors’ translation) would create a greater commitment to studying the content of the various lessons.

Thus, structure-related factors, such as opportunities for interaction, marking work or fixed timeframes, are essential for an ideal online offering because they can support the students’ self-regulation.

### Choice of format from the perspective of educational planning

After changing their perspective to that of an educational planner, the respondents named the following areas as being more suited to the face-to-face format: teaching the compulsory curriculum, practical skills, content with high professional relevance and topics students perceive as difficult. Their preference for this format can be attributed in particular to the opportunity for direct interaction in the form of communication or feedback (4 mentions).

*‘For me a bit of exchange is part of studying, so with both teaching staff and fellow students, um, and I think an online course just simply doesn’t offer that. So, even if there's a chat or something like that, only a fraction of the, the participants use it, because a lot of them just somehow work through it in front of the computer and then do something else as quickly as possible. Um, I think you remember things better when you learn in a group.’* (Interview 2, authors’ translation)

On the other hand, the online format was preferable to face-to-face teaching in large groups (4 mentions) and for topics with a high proportion of theoretical knowledge (e.g. pharmacology; two mentions) or little clinical-practical relevance, e.g. ‘statistical stuff like biometry’ (Interview 1, author’s translation; two mentions).

In addition, as educational planners several respondents would offer online courses as a preparation or supplement to face-to-face courses or as an alternative to face-to-face courses that have a limited number of seats.

### Required behaviour change depending on the format chosen

The interviewees stated that under certain conditions their choice could be influenced.

Switch from *face-to-face* to *online*: Making more of the online formats mandatory in the curriculum – as opposed to voluntary – was named as a prerequisite for someone to switch to this format (2 mentions). Instead of being completely time independent, fixed timeframes should be specified for working through the topics. Another proposal was to integrate blended learning elements into the online format, e.g. in the form of classroom-based sessions in which application examples are used (see also Table 2).

Switch from *online* to *face-to-face*: Educational planners would have to ensure that the face-to-face format takes advantage of the special features of this format, such as enabling direct contact with patients. Also, because the face-to-face format has a fixed time and place, the effort of attending would have to be minimized by optimising the timetable (see also Table 2).

A switch to the face-to-face format was considered to be worthwhile if the group size is small, ‘like these seminars for up to 20 people’ (Interview 4, author’s translation), because a smaller group would promote active participation by the students.

### Personal reasons for choosing a particular format

#### Content-related factors

As regards the motivation for participating in the ECG course, the respondents indicated that the course content was ‘highly relevant’ and that the compulsory curriculum did not include any courses on the topic. For example, in interview 4 the student stated the following:

*‘[... so I think later on as a doctor you should evaluate ECGs, no matter in which discipline you are, you should be able to evaluate the most important things in an ECG, that was my motivation. And because it is not in the regular curriculum, I took it as an elective course.]’* (Interview 4, authors’ translation)

Students experience the relevance of the topic of ECG in the practical phases of their degree. Their own uncertainty and the uncertainty they perceive in others in dealing with different aspects of the topic motivated them to attend the course and examine the subject matter. The respective statements were identical for both course formats and a preference for one format or the other could not be derived from them.

The majority of students (7 out of 10) considered the topic of ECG to be difficult, complex and multifaceted. Several participants stated that the face-to-face course was the more appropriate format for difficult topics.

*‘So if, if I, um, assume that a topic will be very difficult then I would also decide again for the face-to-face course.’* (Interview 10, authors’ translation)

Thus, there was a trend to associate the difficulty of the topic with the choice of format.

### Learning style factors

Nine of the 10 respondents named actions such as notetaking, writing excerpts / summaries, etc., as important additional parts of their personal learning strategy, regardless of the format chosen. Two interviewees showed a genuine aversion to learning on the computer for longer periods (one of them wandered off topic when working on the computer and the other found it too strenuous to read on the monitor).

However, the factors listed in the category cluster ‘Learning style factors’ (learning type, teaching methods, exchange with others, strategic learning planning) had no decisive influence on the choice of format.

### Personal reasons for making a particular decision

#### Online format – Flexible time allocation, repeatability & individual learning speed

The two main reasons for choosing the online format were given as the ability to freely allocate time and the ability to repeat the subject matter as often as desired. The greater flexibility of the online course, e.g. with regard to learning speed, was also rated positively.

*‘[...] the nice thing about the online course is [...] that I can listen to it as often as I want. If I just noticed that parts of it were going too fast and, um, ( ) I can pause it now, I can write things down, I can carry on again, I can listen to a lecture twice, um, ( ) just as I like. One is simply more flexible.’* (Interview 8, authors’ translation)

Corresponding with the statements regarding the online format, the lack of time flexibility and the greater effort of participating (e.g. travel expenses) were rated negatively for face-to-face courses.

#### Face-to-face course - external control, commitment and interaction with lecturers

The negative side of flexible time allocation, however, was also apparent, because the two students who had discontinued the online course indicated the lack of a fixed time (‘lack of pressure’) as the reason for stopping.

*‘[...] so I did the online course last year, but at lesson six, what with one thing and another, you plan to do it, but then if there isn’t any pressure on you, um, then it was exam period and then at some point I abandoned it.’* (Interview 4, authors’ translation)

This statement, characterizing the lack of pressure in the online course as a disadvantage is noteworthy with regards to the remarks of three participants in the face-to-face course, who perceived the prevailing degree of external control (‘compulsory attendance’), accompanied by a higher degree of commitment, as a positive aspect.

*‘[...] and then I thought, well, for, for someone like me who sometimes simply does not have the discipline to stay on the ball completely without, um, the external control, such as appearing every week, for example, [...] in that case a face-to-face course simply makes more sense and so that’s what I did.’* (Interview 9, authors’ translation)

In addition, three participants in the face-to-face course rated the opportunity for direct interaction with the lecturer in the form of questions and discussions as a key decision-making criterion for their choice of that format. This interaction would allow a more comprehensive understanding than the online course:

*‘[...] l enjoyed having some exercises here and there [...] that we talked about it in the seminar, that we received feedback [...].’* (Interview 10, authors’ translation)

Last but not least, personal recommendations by older students played a role. On the one hand these students stressed the teaching activities of the responsible lecturer (2 mentions) and, on the other hand, they warned of the higher risk of discontinuing the online course, because of its non-committal character.

The choice of format was therefore primarily based on the weight students placed on the factors ‘flexible time management, repeatability and individual learning speed’ of the online format versus ‘more external control and commitment, as well as interaction with lecturers’ in the face-to-face format. These factors and their relative weighting were influenced by personal experience or were passed on by older fellow students.

## Discussion

As the analysis of our interviews shows, from the students’ perspective the aspects of time and location flexibility, which are always mentioned in connection with online courses, need to be considered in a nuanced way: although access to the learning content *regardless of the time of day* was rated positively by all students, the *lack of a time limit* for the availability of the learning content was viewed critically. Thus, two interviewees named the lack of a fixed timeframe (e.g. by when lessons had to be completed) and the associated lack of pressure or extrinsic motivation as the main reason why they had discontinued the online ECG course and why they subsequently decided to take the face-to-face course. The non-binding nature of the online course therefore requires the students to have a high degree of initiative and discipline in order to actually complete the course. Despite the perceived high professional relevance of the topic ECG and consequently of the learning content, many students do not achieve this level of intrinsic motivation and self-regulation (as is indicated as well by the low participation rate in the final examination), possibly because the high relevance is counteracted by competing or more extrinsically motivated demands on the medical students (e.g. mandatory attendance at face-to-face instruction and compulsory appointments). Strengthening the ability to learn in a self-regulated way has long been central to curricular development in medicine [16], although studies emphasise that it still needs to be enhanced [17].

The students viewed e-learning’s flexibility of location in a similarly differentiated way to time flexibility. It is the obligation to be *present* on a particular day at a fixed time at a fixed location that results in face-to-face courses requiring a higher degree of commitment both concerning adequate preparation of the learning content and willingness to complete the course. This finding is strengthened by the additional online survey: students indicated that preference is given to *on-site* teaching, even though all respondents have the necessary technical prerequisites to participate in online courses.

In addition, because of the personal feedback students received in the face-to-face course we assume that they perceived themselves as being more self-efficacious than those in the online course who did not receive individual feedback. This assumption is supported by research showing individual feedback to increase students’ self-efficacy [18, 19]. In particular the social and emotional support provided by lecturers and students by means of direct feedback can help to strengthen learners’ confidence in their own skills – even though self-reported estimates of competence are an unreliable measure of competence [20]. The experienced self-efficacy itself leads to more self-motivation, effort and perseverance [21, 22]. If nothing else, these are important factors for successfully completing a course. Notably, two interviewees named a lack of perseverance as a decisive reason why they had discontinued the online ECG course. Motivation has also been shown to be an important factor for students’ performance in online courses [23]. Whether or to what extent e-learning offerings can promote the self-efficacy experienced by participants is therefore an important question when establishing effective learning formats in this field.

The interaction between learners, teachers and learning contents has been described as a central element in the learning process [24]. The evaluation of our interviews suggests this very aspect to be a decisive strength of face-to-face instruction that cannot be replaced one-to-one by online courses. The opportunity to address the lecturer directly and obtain an immediate answer was considered to be a crucial advantage of this format. Also, in the case of complex topics the exchange with fellow students in face-to-face courses is experienced as beneficial. The extent to which these possibilities for interactions are integrated into online courses is thus a guiding question for the development of e-learning formats. However, known types of online interaction such as forums, chats or virtual pinboards do not fully meet this challenge and as far as possible direct (face-to-face) forms of communication are needed.

Limitations of our study concern the small sample size, limiting the generalisability of the results. Even though similar statements were made by several interviewees, additional interviews with further students might have contributed to a broader variety of statements. Therefore, the possible range of different assertions was probably not fully exhausted.

The inclusion of students who had discontinued the online course and then later participated in the face-to-face course may have given rise to a potential bias in the sense of a distortion in favour of face-to-face courses. However, the analysis of the interviews showed that one of these students explicitly named the advantages of the online course and the other could easily have imagined participating in the online course again. This group also provided exclusive information based on their experience with both formats. Furthermore, in particular the very students who discontinue a course may provide valuable information on reasons for withdrawal.

Pass / fail rates for the examination are not routinely recorded for the two courses and are therefore not available to us. None of the interviewees gave any indication that the examination results influenced the choice of format. This makes a respective bias unlikely.

We are unable to comment on the relative efficacy of the two formats from the point of view of assessment performance, as this was not a stated aim of this study.

## Conclusions

Despite the limitations mentioned above, our results provide first indications that the following three aspects could be of particular interest in the future development of online courses in order to better meet students’ needs:

### Extrinsic motivation – Create both fixed timeframes for working on topics and external support of self-regulation

Because students’ intrinsic motivation alone does not prevent them from discontinuing an online course, external support programmes are also required in order to maintain self-motivation, effort and perseverance in equal measure. Therefore, instead of the learning content being available for an indefinite time at any location we recommend specifying fixed timeframes for working through topics and integrating face-to-face teaching at fixed times. As a further tool, automatic exclusion from the course if a deadline is not met is conceivable as an *external aid for self-regulation* if, for example, a corresponding lesson has not been completed by a pre-determined time.

### Intrinsic motivation – Feedback to students

Individual feedback is crucial for successful completion of online courses. Feedback from lecturers to students promotes self-efficacy and intrinsic motivation. One conceivable option to integrate personal feedback into an online course would be in the form of submitted and corrected homework assignments, for example. Learning controls during or at the end of a learning unit, for example in the form of embedded multiple-choice questions on previously presented material, could also increase attention and motivation.

### Preference of formats that enable at least some personal interaction with the lecturer

Building on the previous point, the statements of our interviewees indicate that face-to-face courses cannot be replaced one-to-one by online courses as regards the opportunities for interaction between lecturers and students or between students. However, because interaction – especially in the form of feedback [25] – is a key factor for learning success, we recommend hybrid forms with a minimum of personal interaction instead of purely online courses.

The points described above thus mean a shift from purely online courses to formats that are more strongly interspersed by elements from face-to-face courses (blended learning), at least for difficult or important subject matters. This proposed change is supported by the high level of agreement in the additional questionnaire that although e-learning formats are a good supplement to face-to-face instruction they are not an adequate replacement for theory-oriented face-to-face courses when it comes to students’ needs.

To conclude, we believe that the knowledge we gained from this study is sufficiently valuable to be taken into consideration when developing future e-learning tools in our faculty.

## Abbreviations

ECG: Electrocardiogram
SPSS: Sammeln, prüfen, sortieren, subsumieren, i.e. collect, check, sort, prioritise
